# Atrial late gadolinium enhancement on MRI relates to the electrophysiological substrate of persistent atrial fibrillation

**DOI:** 10.1186/1532-429X-17-S1-O22

**Published:** 2015-02-03

**Authors:** Stephanie Clement-Guinaudeau, Michel Montaudon, François Laurent, Pierre Jaïs, Hubert Cochet

**Affiliations:** 1 Hôpital Cardiologique Haut-Lévèque, Pessac, France

## Background

The mechanisms responsible for atrial fibrillation (AF) maintenance are poorly understood. We studied the relationship between focal atrial fibrosis as assessed with late gadolinium enhanced (LGE) cardiac magnetic resonance (CMR), and the electrophysiological substrate of persistent AF as assessed with body surface potential mapping (BSM).

## Methods

We studied 41 patients with persistent AF (6 women, age 56+/-12 years). Patients underwent LGE CMR using an respiratory navigated and inversion recovery prepared 3D turbo FLASH sequence with fat saturation (pixel size 1.25×1.25×2.5mm), as well as non-invasive BSM during atrial fibrillation using a 256-electrode vest, enabling real-time panoramic mapping of atrial electrical activation. On CMR images, the bi-atrial wall was manually segmented and LGE was quantified using an adaptive histogram thresholding algorithm. The result was both a global quantification of LGE on the left atrial wall expressed in % of the wall and categorized according to Utah classification (I: <10%, II: 10-20%, III: 20-30%, IV: >40%), and a patient-specific 3D map displaying LGE distribution on both atria. On BSM data, phase mapping was applied to visualize electrical activation, and atrial fibrillation drivers were defined as rotors (phase singularities) lasting more than 200ms. The sites exhibiting high rotor activity were defined as driving regions and targeted by catheter ablation, with the acute endpoint of AF termination. We assessed the relationship between global LGE burden and patients' clinical characteristics, electrophysiological characteristics, and acute procedural success. In a subset of 12 patients, CMR and BSM data were registered to assess the spatial relationship between LGE and AF drivers.

## Results

Mean AF duration was 8+/-6 months. On CMR, the mean LGE burden on the left atrium was 22+/-5.9%. According to Utah classification, there were no class I, 16 class II, 21 class III, and 4 class IV. On BSM, the mean number of driving regions targeted by ablation was 4.3+/-1.7 per patient (range 2 to 8). AF could be terminated by ablation in 28/41 (68%) patients. LGE was positively related to AF duration (R=0.59, P=0.01) and to the number of driving regions (R=0.51, P=0.03). LGE did not relate to age (R=0.17, P=0.15) or gender (R=0.18, P=0.15). LGE burden and the number of driving regions were both inversely related to acute procedural success (R=-0.53, P=0.02 and R=-0.58, P=0.01). In the subset of 12 patients with registered CMR and BSM data, visual analysis of rotor trajectories showed a clustering around LGE areas (Figure).

**Figure 1:**
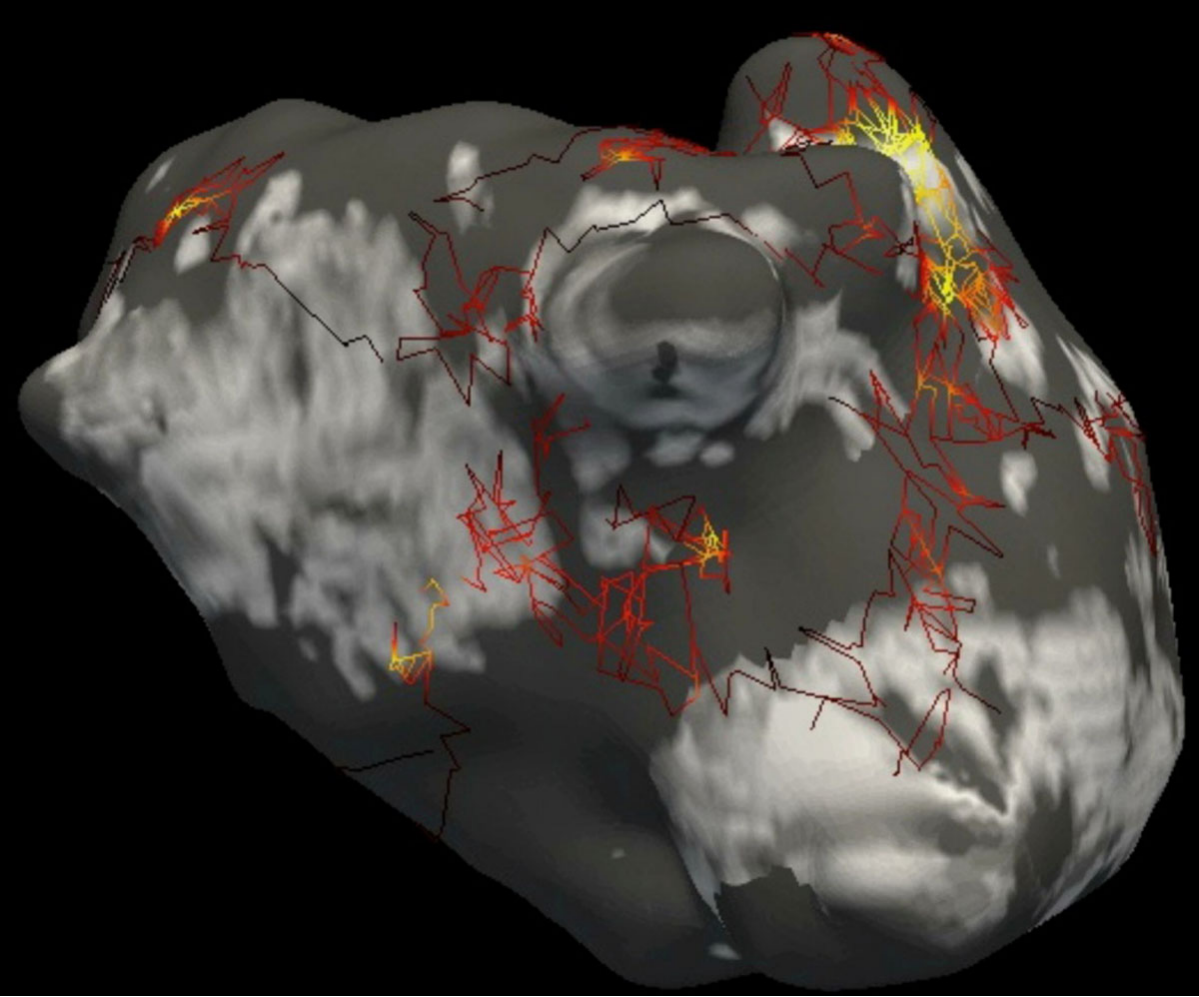
Rotor trajectories registered to late gadolinium enhancement distribution in a 62 year-old patient with persistent atrial fibrillation. Rotor trajectories are color-coded according to the persistence of the rotor at each point of its trajectory, in order to visualize anchoring sites responsible for atrial fibrillation maintenance. These driving regions are consistently observed at the borders of LGE areas.

## Conclusions

Focal atrial fibrosis assessed by LGE CMR is associated to persistent AF duration and to the number of regions driving the arrhythmia, i.e. a surrogate for AF complexity. In patients undergoing catheter ablation targeting AF drivers, the extent of focal fibrosis is inversely related to acute procedural success. Futhermore, atrial fibrillation drivers are clustered along the borders of fibrotic areas.

## Funding

N/A.

